# Dextrocardia and asplenia in situs inversus totalis in a baby: a case report

**DOI:** 10.1186/1752-1947-8-408

**Published:** 2014-12-05

**Authors:** Abnish Kumar, Manoj Kumar Singh, Neeraj Yadav

**Affiliations:** Department of Pediatrics, S. N. Medical College, Agra, Pin Code-282002 India

**Keywords:** Dextrocardia, Electrocardiogram, Situs inversus

## Abstract

**Introduction:**

Situs inversus with dextrocardia is the complete inversion of position of the thoracic and abdominal viscera. It may be isolated or associated with malformations, especially cardiac and/or alimentary. It may be discovered in infancy because of associated anomalies but often remains asymptomatic and discovered incidentally in adult life. Only a small number of cases have been reported from India.

**Case presentation:**

We report the case of a 7-month-old Indo-Aryan baby girl found to have dextrocardia with situs inversus totalis who presented with fever, cough and respiratory distress. A chest X-ray showed her heart in the right hemithorax with the cardiac apex pointing towards the right. The findings of an electrocardiogram and echocardiography confirmed the location of her heart in the right hemithorax and an abdominal sonogram showed her liver and gall bladder in midline of her abdomen whereas her stomach was located more towards the right side, her spleen was absent.

**Conclusions:**

Situs inversus totalis, although a rare condition, should be sought for when clinical and radiologic findings indicate dextrocardia, especially as it may be an incidental finding. Doctors should encourage routine medical examination for their patients which could help identify this anomaly, thereby preventing wrong diagnosis and possibly death due to delay in management.

## Introduction

Situs describes the position of the cardiac atria and viscera [1, 2]. Situs solitus is the normal position, and situs inversus is the mirror image of situs solitus. Situs inversus with dextrocardia is termed situs inversus totalis because the cardiac position, as well as abdominal viscera, is the mirror image of the normal anatomy. Situs inversus is a rare condition. A few cases of situs inversus totalis have been described in the literature. We report a case of situs inversus totalis.

## Case presentation

A 7-month-old Indo-Aryan baby girl of non-consanguineous parents was admitted to our emergency department with the complaint of cough and cold for 5 days, fever for 2 days, fast breathing with chest retraction for 1 day and refusal to feed for 1 day. There is no history of a similar episode in the past. There was no history of diabetes, cocaine use or any other drug intake in her mother.

On examination her vitals were temperature of 37.44°C (99.4°F) of 76/minute, heart rate of 126/minute, and blood pressure of 84/60mmHg in her right upper arm in supine position. A cardiovascular system examination showed visible apex beat in right fifth intercostal space in midclavicular line. There was cardiac dullness on her right side and pansystolic murmur was heard at the apex. Heart sounds were louder on the right side of her chest. Abdominal examination showed no palpable organomegaly but on percussion liver dullness was on left side and tympanic note was present over right hypochondrium.

**Figure 1:**
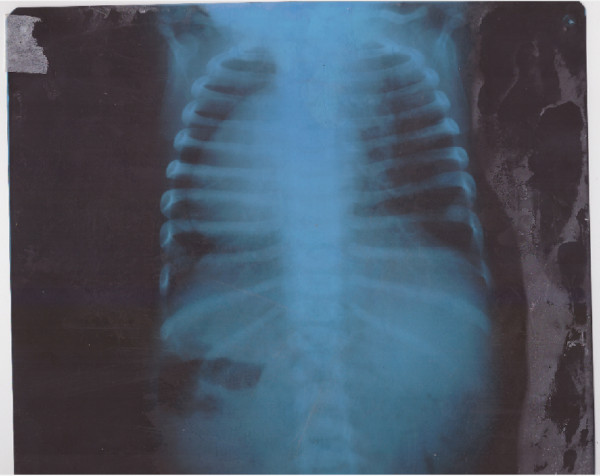
**Chest X-ray (posteroanterior view) showing heart in the right hemithorax.**

**Figure 2:**
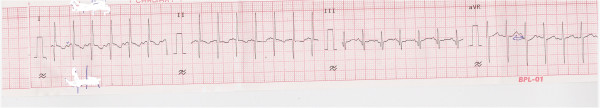
**Electrocardiography showing inverted p wave in lead I.**

**Figure 3:**
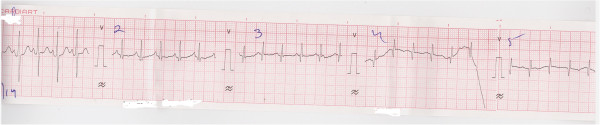
**Electrocardiography showing reverse progression of R wave in leads V1 to V6.**

**Figure 4:**
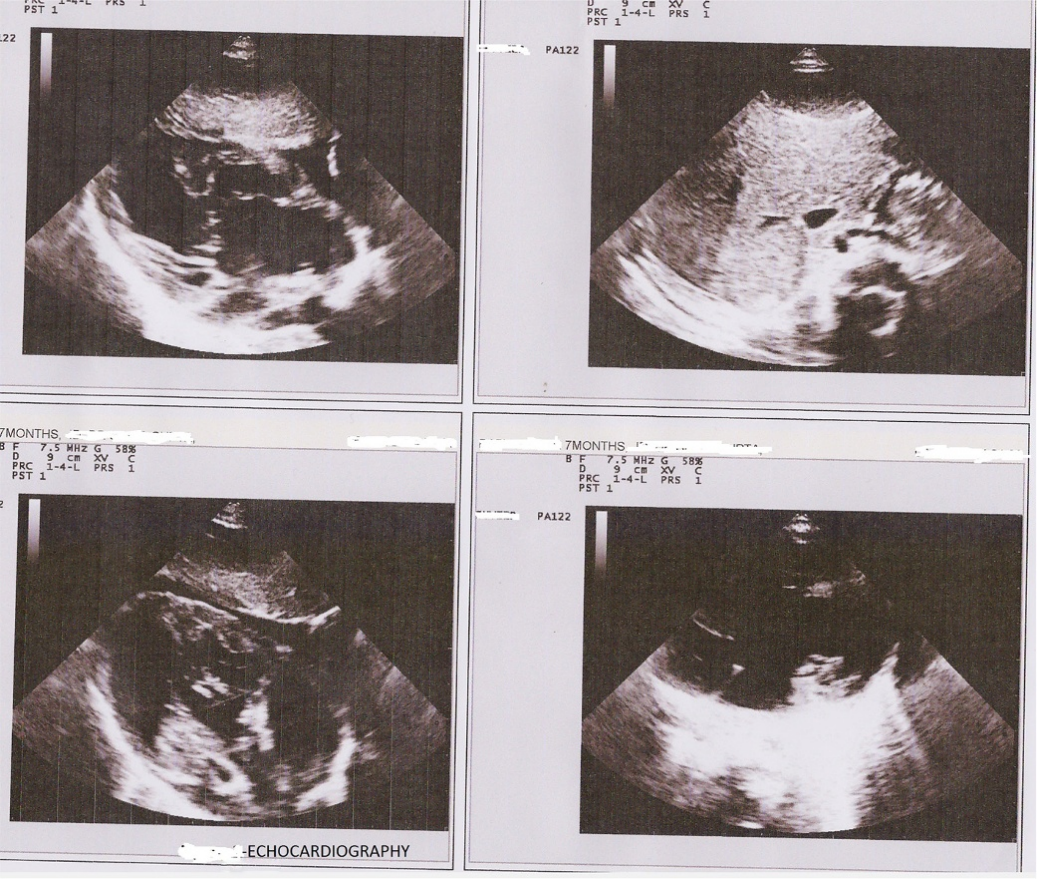
**Echocardiography showing complex pattern of heart disease.**

A chest X-ray posteroanterior view (Figure 1) showed her heart in the right hemithorax with the base to apex axis pointing towards the right. Her lung fields were clear. Her thoracic cage was normal. Electrocardiography (ECG) showed inverted p wave in lead I and positive QRS complex (Figure 2), positive p wave in lead avR, inverted p wave in avL and reverse progression of R wave in leads V1 to V6 (Figure 3); ECG tracing with reversed limb leads revealed positive p in lead I. Echocardiography demonstrated dextrocardia, inferior vena cava (IVC) and aorta on right side, dilated right ventricle and right atrium, large ostium primum atrial septal defect (ASD), large ventricular septal defect (VSD) at perimembranous position with bidirectional shunt, transposition of great arteries (TGA), severe aortic regurgitation (AR) and severe subvalvular pulmonary stenosis (PS; Figure 4).

**Figure 5:**
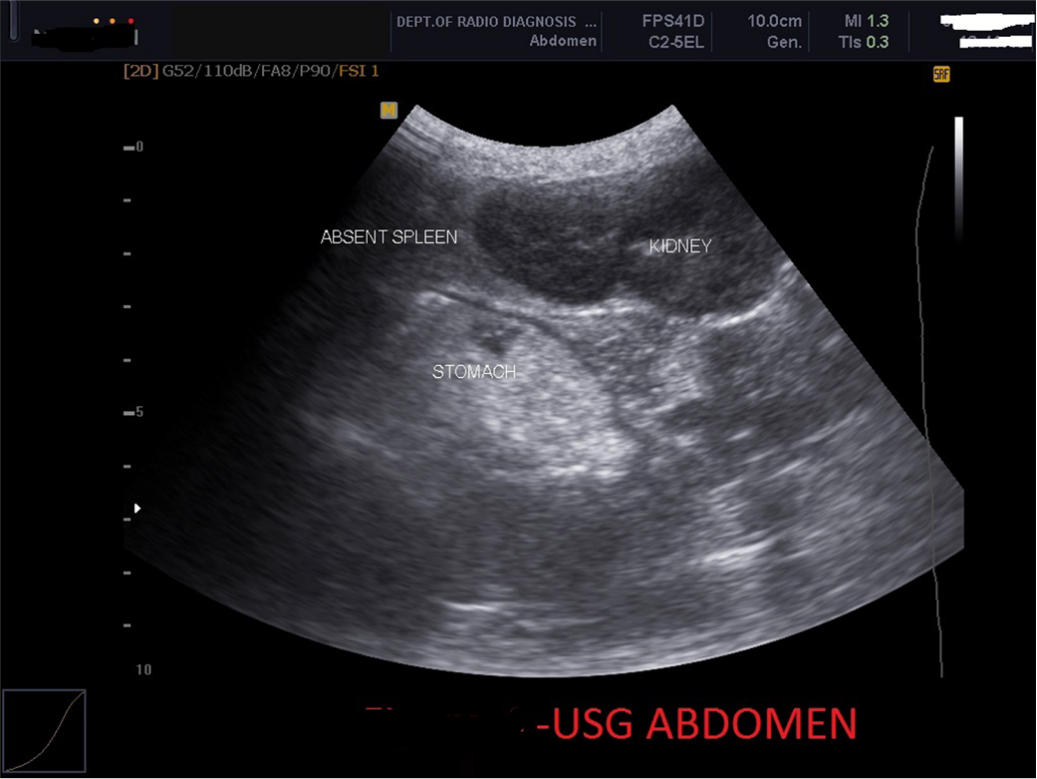
**Abdominal ultrasound showing absent spleen.**

Abdominal ultrasound revealed a symmetrical midline liver and stomach towards midline, IVC and aorta on right side, absent spleen and normal kidneys (Figure 5).

## Discussion

Situs inversus is a rare congenital anomaly reported to occur in 1 in 8000 to 1 in 25,000 patients [3]. No racial predilection exists for situs inversus. The male-to-female incidence is 1:1. The arrangements of the position of the abdominal viscera in dextrocardia may be normal (situs solitus), reversed (situs inversus), and indeterminate (situs ambiguous or isomerism) in 32 to 35%, 35 to 39% and 26 to 28% of cases respectively [4].

In our patient situs inversus was associated with asplenia and dextrocardia. Cardiac anomalies identified on echocardiography were ASD, VSD, AR, PS and TGA. Both her kidneys were normal. In the vascular anomaly IVC and aorta were both on her right side. Dextrocardia with a normal abdominal situs has a high incidence of associated congenital cardiac anomalies including among others, transposition of the great vessels and ASDs [5] and VSDs [6] in 90 to 95% of cases. However, dextrocardia with situs inversus is associated with a lower incidence of congenital heart disease (0 to 10%) as was the case in our patient. Presentation of cause varies depending on associated malformation [1–3]. Situs inversus may be associated with other congenital anomalies such as duodenal atresia, asplenism, multiple spleens, ectopic kidney, horseshoe kidney and various pulmonary and vascular abnormalities. Situs inversus totalis that is associated with primary ciliary dyskinesia is known as Kartagener syndrome [7, 8]. Patients with primary ciliary dyskinesia have repeated sinus and pulmonary infections [7, 9]. Frequent pulmonary infections often result in bronchiectasis, which predominantly affects the lower lungs. Typically, persons having situs inversus with dextrocardia without other congenital anomaly have a normal life expectancy and have a similar risk of getting acquired disease as that of other persons of the same age and sex group. In the rare instances of cardiac anomalies, life expectancy is reduced, depending on the severity of the defect [10]. The recognition of situs inversus is also important for preventing surgical mishaps that result from the failure to recognize reversed anatomy or an atypical history. For example, in a patient with situs inversus, cholecystitis typically causes left upper quadrant pain, and appendicitis causes left lower quadrant pain. Cardiac situs is determined by the atrial location. In situs inversus, the morphologic right atrium is on the left, and the morphologic left atrium is on the right. The normal pulmonary anatomy is also reversed so that the left lung has three lobes and the right lung has two lobes. In addition, the liver and gallbladder are located on the left, whereas the spleen and stomach are located on the right. The remaining internal structures are also a mirror image of the normal. In a study of 111 cases, Merklin and Varano classified cases of situs inversus into: (a) complete situs inversus; (b) dextrocardia with situs solitus; (c) partial situs inversus; (d) dextroposition of the heart; and (e) levocardia [11]. Although the exact cause is unknown, dextrocardia has been linked with several factors including autosomal recessive gene with incomplete penetrance, maternal diabetes, cocaine use, and conjoined twinning [12–14]. Diagnosis of dextrocardia is usually confirmed by several modalities which include chest radiography, ECG, echocardiography, computed tomography, magnetic resonance imaging and abdominal ultrasonography. Echocardiography is one of the modalities for making the diagnosis. Of interest, this patient had situs inversus totalis with multiple cardiac lesions ASD, VSD, AR, PS and TGA. This case is reported because of the situs inversus, dextrocardia and asplenia with early symptomatic presentation due to complex pattern of cardiac malformation.

## Conclusions

Dextrocardia with situs inversus is a rare congenital malformation that must be fully evaluated when noticed because in rare instances it may result in fatal outcome. There is need for a complete and elaborate diagnostic work up of suspected cases by various imaging modalities so that they are not missed. Surgeons, radiologists and radiographers should look out for this anomaly during preoperative and surgical management of their patients. Doctors should encourage routine medical examination for their patients which could help identify this anomaly, thereby preventing wrong diagnosis and possibly death due to delay in management.

## Consent

Written informed consent was obtained from the patient’s legal guardian(s) for publication of this case report and any accompanying images. A copy of the written consent is available for review by the Editor-in-Chief of this journal.

## Abbreviations

AR: Aortic regurgitation
ASD: Atrial septal defect
ECG: Electrocardiography
IVC: Inferior vena cava
PS: Pulmonary stenosis
TGA: Transposition of great arteries
VSD: Ventricular septal defect.
